# Mutation of *gdpS* gene induces a viable but non-culturable state in *Staphylococcus epidermidis* and changes in the global transcriptional profile

**DOI:** 10.1186/s12866-022-02708-6

**Published:** 2022-12-01

**Authors:** Tao Zhu, Wei Wang, Han Wang, Yanfeng Zhao, Di Qu, Yang Wu

**Affiliations:** 1 Department of Medical Microbiology and Immunology, Wannan Medical College, Wuhu, 241002 People’s Republic of China; 2 Department of Pharmacy, Wannan Medical College, Wuhu, 241002 People’s Republic of China; 3 Department of Laboratory Medicine, the Second Affiliated Hospital of Nanjing Medical University, Nanjing, 210011 People’s Republic of China; 4 Key Laboratory of Medical Molecular Virology (MOE/NHC/CAMS), Department of Medical Microbiology and Parasitology, School of Basic Medical Sciences, Shanghai Medical College of Fudan University, Shanghai, 200032 People’s Republic of China

**Keywords:** *Staphylococcus epidermidis*, *gdpS*, Viable but nonculturable, Low temperature, Transcriptional profile

## Abstract

**Background:**

In the genome of staphylococci, only the *gdpS* gene encodes the conserved GGDEF domain, which is the characteristic of diguanylate cyclases. In our previous study, we have demonstrated that the *gdpS* gene can modulate biofilm formation by positively regulating the expression of *ica* operon in *Staphylococcus epidermidis*. Moreover, this regulation seems to be independent of the c-di-GMP signaling pathway and the protein-coding function of this gene. Therefore, the biological function of the *gdpS* gene remains to be further investigated.

**Results:**

In the present study, it was observed that mutation of the *gdpS* gene induced *S. epidermidis* to enter into a presumed viable but nonculturable state (VBNC) after cryopreservation with glycerol. Similarly, when moved from liquid to solid culture medium, the *gdpS* mutant strain also exhibited a VBNC state. Compared with the wild-type strain, the *gdpS* mutant strain autolyzed more quickly during storage at 4℃, indicating its increased susceptibility to low temperature. Transcriptional profiling analysis showed that the *gdpS* mutation affected the transcription of 188 genes (92 genes were upregulated and 96 genes were downregulated). Specifically, genes responsible for glycerol metabolism were most markedly upregulated and most of the altered genes in the mutant strain are those involved in nitrogen metabolism. In addition, the most significantly downregulated genes included the *betB* gene, whose product catalyzes the synthesis of glycine betaine and confers tolerance to cold.

**Conclusion:**

The preliminary results suggest that the *gdpS* gene may participate in VBNC formation of *S. epidermidis* in face of adverse environmental factors, which is probably achieved by regulating expression of energy metabolism genes. Besides, the *gdpS* gene is critical for *S. epidermidis* to survive low temperature, and the underlying mechanism may be partly explained by its influence on expression of *betB* gene.

**Supplementary Information:**

The online version contains supplementary material available at 10.1186/s12866-022-02708-6.

## Introduction

The GGDEF domain is characteristic of diguanylate cyclases (DGCs) that are responsible for synthesis of bis-(3′, 5′)-cyclic dimeric GMP (c-di-GMP). The domain is named after its conserved signature motif Gly-Gly-Asp-Glu-Phe. C-di-GMP is a ubiquitous and important second messenger in bacteria [1, 2]. It has been implicated in a growing number of physiological processes, including biofilm formation, motility, virulence, cell cycle and differentiation [2, 3]. Generally, low levels of the intracellular second messenger are related to planktonic growth, whereas increased concentrations favor surface attachment and biofilm formation [3, 4].

Intracellular pools of c-di-GMP fluctuate dynamically in response to internal or external stimuli. This is achieved through the antagonistic activities of DGCs and c-di-GMP-specific phosphodiesterases (PDEs). Two classes of structurally and mechanistically distinct PDEs, which typically contain EAL and HD-GYP domains respectively, have been described. The EAL domain catalyzes the cleavage of c-di-GMP to generate the linear molecule 5′-phosphoguanylyl-(3′-5′)-guanosine (pGpG). The HD-GYP domain is responsible for degradation of c-di-GMP into two molecules of GMP [3, 5, 6]. It has been demonstrated that c-di-GMP exerts its regulatory function by binding to a wide variety of effectors including kinases or phosphorylases, transcription factors, PilZ domain proteins, degenerate DGCs or PDEs, and riboswitches [7].

Biofilm formation is a key virulence determinant for many microorganisms that cause chronic and device-associated infections [8, 9]. The bacteria enclosed within the biofilm are recalcitrant to the hosts’ immune response and antimicrobial agent clearance, so that medical interventions such as surgery are often required to treat infected tissues and/or remove indwelling devices[10, 11]. In a number of *Staphylococcus epidermidis* and *Staphylococcus aureus* strains, the major component of biofilm matrix is the exopolysaccharide termed polysaccharide intercellular adhesin (PIA) or polymeric N-acetyl-glucosamine (PNAG). The synthesis of PIA/PNAG is encoded by the *ica* operon [12, 13].

Only one gene encoding the GGDEF domain protein, designated as *gdpS,* is present in sequenced staphylococcal genomes. Moreover, neither genes encoding EAL or HD-GYP domain proteins nor those encoding PilZ domain proteins are found in their genomes [14]. Holland et al. and we have investigated the role of the *gdpS* gene in biofilm formation in *S. epidermidis* [15]. It was found that *gdpS* can promote biofilm formation by elevating *ica* operon transcription. When exploring the mechanism by which *gdpS* regulates *ica* transcription, unexpectedly, the regulation was found to be independent of c-di-GMP synthesis. Mutagenesis of the GGDEF domain did not abolish the capacity of *gdpS* to restore the biofilm defect of the *gdpS* mutant. Furthermore, heterologous DGC expressed *in* *trans* failed to complement the *gdpS* mutant, and recombinant GdpS protein exhibited no DGC activity in vitro [15, 16]. These observations indicated that the *gdpS* gene might represent remnants of the c-di-GMP signaling pathway.

Indeed, this speculation is also supported by the mechanistic insight into the non-coding role of *gdpS* on *spa* gene expression in *S. aureus*, as reported by Shang [14]. They found that the RNA transcript of *gdpS* can directly bind to the 5’ UTR of *sarS* mRNA, leading to stabilization of the latter [17]. The global regulatory locus *sarS* is a *sarA* homolog and can activate transcription of *spa* [18]. Inspired by these findings, through site-mutagenesis of start codon and by complementation experiments, we have suggested that *gdpS* in *S. epidermidis* may also modulate biofilm formation at the post-transcriptional level [16]. However, the protein product of the *gdpS* gene has also been detected in *S. aureus* and *S. epidermidis* through Western blot analysis [16, 17]. Therefore, it is worthwhile to explore the biological function of the *gdpS*-encoding protein. In the present study, we sought to investigate the physiological role of *gdpS* by comparing phenotypic and transcriptional profiling variations between *S. epidermidis* strain 35984 M and its *gdpS* mutant derivative.

## Results and discussion

### Mutation of *gdpS *induces *S. epidermidis* to enter into a VBNC state upon low temperature and osmotic pressure

**Fig. 1:**
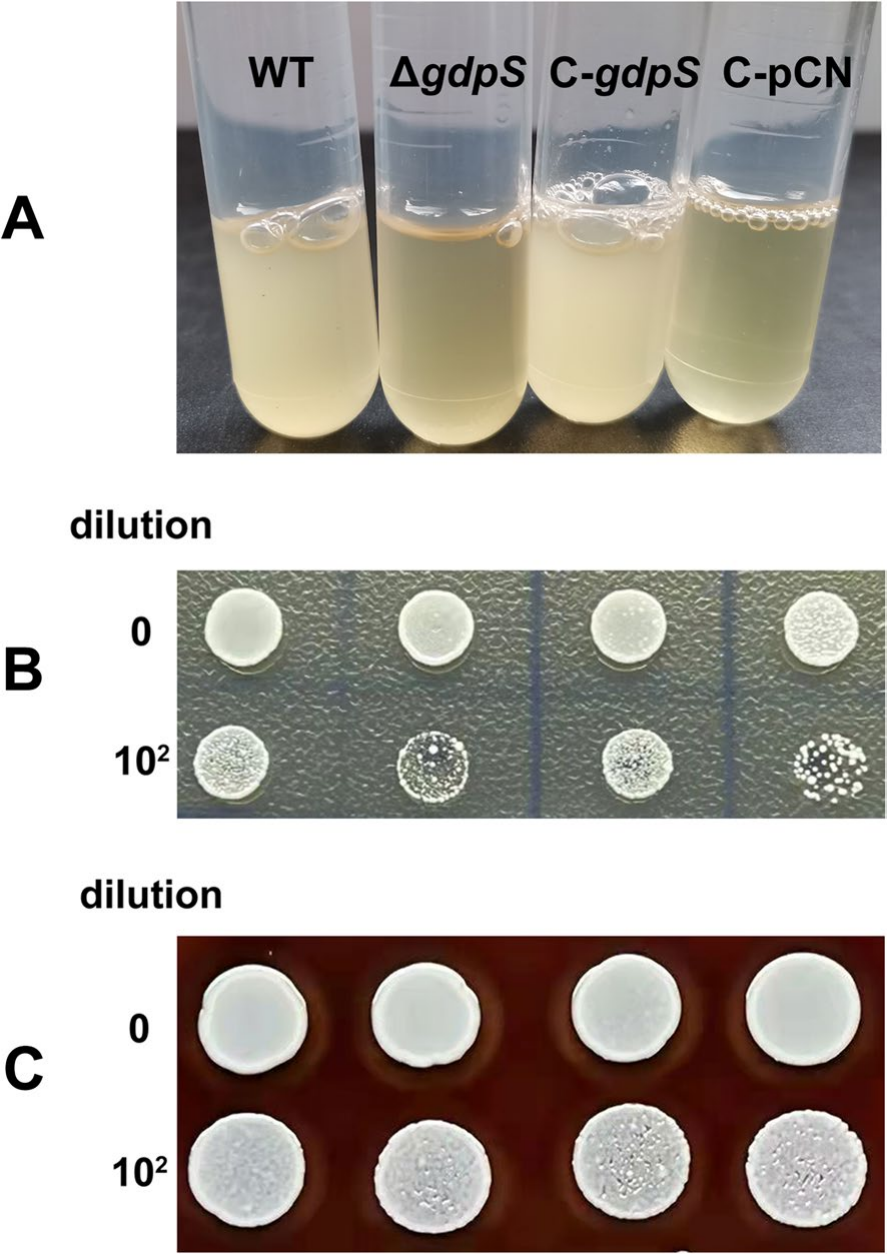
Mutation of *gdpS* induces *S. epidermidis* to enter into a VBNC state after cryopreservation. The cryopreserved *S. epidermidis* strains were thawed, inoculated directly into liquid TSB medium (**A**) at a dilution of 1:100, and then incubated with agitation at 37 °C for over 16 h. Meanwhile, the thawed cultures and their corresponding dilutions were spotted onto TSB agar plate (**B**) and Columbia blood agar plate (**C**) respectively, and cultivated at 37 °C for 24 h. WT: the wild type strain, Δ*gdpS*: the *gdpS* mutant strain, C-*gdpS*: the *gdpS* mutant strain complemented with the native *gdpS* gene, C-pCN: the *gdpS* mutant strain complemented with the empty vector pCNcat. The experiment was repeated at least three times, and a representative figure is shown

When we reactivated the *gdpS* mutant strain that has been preserved at -80 °C with glycerol as cryoprotectant, obvious growth retardation was observed in comparison with the wild-type strain. As shown in Fig. 1A, when frozen cultures were inoculated into TSB medium at a dilution of 1:100 and incubated at 37 °C with an agitation of 200 rpm for more than 16 h, subcultures of the wild-type and complementation strains exhibited significant turbidity, whereas those of the *gdpS* mutant and the empty vector control strains grew poorly. To determine whether this difference in reactivation is caused by the loss of cell viability after freezing and during frozen storage, both thawed cultures of the *gdpS* mutant strain and its parent strain were spotted onto the TSB agar and Columbia blood agar plates respectively, and cultivated for 24 h. As illustrated in Fig. 1B, when grown on the TSB agar plate, the *gdpS* mutant indeed formed significantly fewer colonies than the wild-type strain. However, when grown on the Columbia blood agar plate, there were no obvious differences in colony formation between the *gdpS* mutant strain and the wild-type strain. The same is true for the complementation strain and the empty vector control strain (Fig. 1C). Moreover, no differences in growth curves were detected between the *gdpS* mutant strain and the wild-type strain after they were fully reactivated.

These results indicated that a sub-population of the *gdpS* mutant strain after frozen storage cannot be cultured in TSB medium although they are alive, which corresponds to the concept of viable but nonculturable (VBNC) cells. Since its first proposal in 1982, many bacterial species have been found to exist in a VBNC state. VBNC cells are characterized by the loss of culturability on routine agar, which hampers their detection by conventional plate count techniques. Although it is controversial that entering VNBC state may be a general strategy adopted by bacteria to survive unfavorable conditions, this leads to an underestimation of total viable cells in environmental and clinical samples, and thus poses a threat to public health [19]. Exposure to various stresses can induce the VBNC state. One of the most frequent inducing factors is low temperature [20, 21], the condition that triggered the presumed VBNC state of the *gdpS* mutant strain in the present study. In addition, previous studies have shown that bacteria in the VBNC state could be resuscitated by rich medium. Our study indeed observed that culturability of the *gdpS* mutant after cryopreservation could be recovered by the nutrient-rich Columbia blood agar. Therefore, we speculate that mutation of *gdpS* can induce *S. epidermidis* to enter into a presumed VBNC state upon low temperature challenge.

**Fig. 2:**
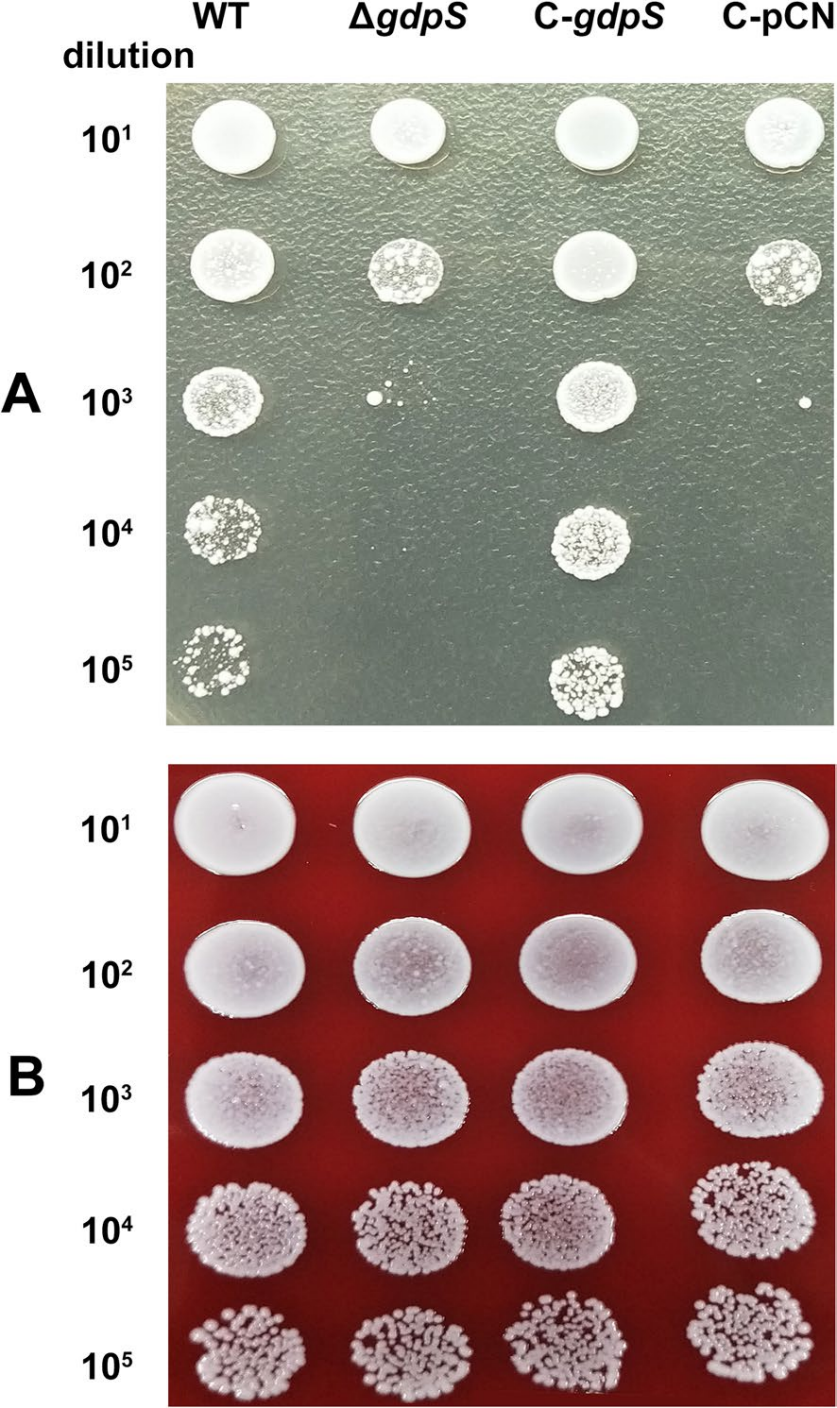
Mutation of *gdpS* induces *S. epidermidis* to enter into a VBNC state under osmotic pressure. All the cryopreserved *S. epidermidis* strains were resuscitated on Columbia blood agar plate and single colonies were inoculated into liquid TSB medium for overnight culture. The overnight cultures were subcultured 1:100 and grown to the exponential phase with identical OD_600_ values in fresh TSB medium, and then serially diluted (1:10), spotted onto TSB agar plate (**A**) and Columbia blood agar plate (**B**) for cultivation, respectively. The experiment was repeated at least three times, and a representative figure is shown

More intriguingly, we were surprised to find that when the *gdpS* mutant was cultured in liquid TSB medium until the mid-exponential phase, and then serially diluted and spotted onto TSB agar, its culturability was significantly reduced compared to the wild-type strain (Fig. 2). One of the key differences between the liquid and solid culture media used here is a change of osmotic pressure. It is thus rational to deduce that osmotic pressure may also trigger the VBNC state of the *gdpS* mutant.

### Mutation *of gdpS *increases the susceptibility of *S. epidermidis *to low temperature

**Fig. 3:**
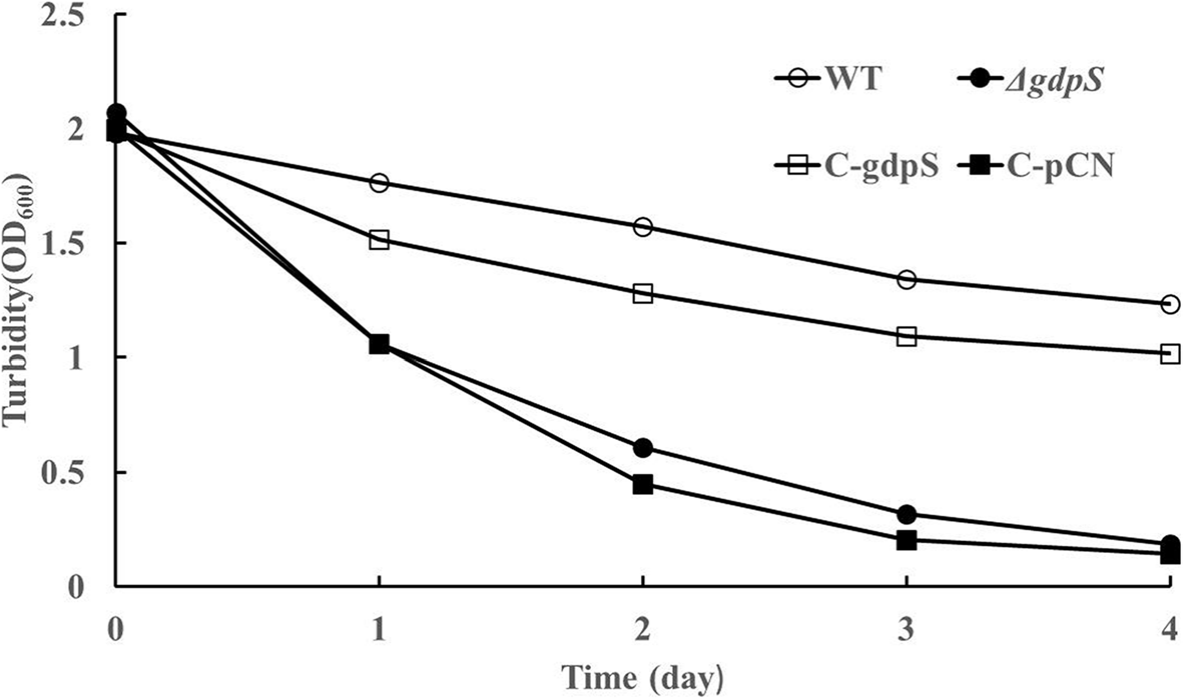
Susceptibility of the Δ*gdpS* strain to low temperature. All the *S. epidermidis* strains were inoculated in fresh TSB medium and grown to logarithmic phase (4 h; OD_600_ = 2) at 37 °C. The cultures were then placed at 4 °C, and the turbidity was measured every day at 600 nm. The experiment was repeated at least three times, and representative curves are shown

Subsequently, to investigate whether the *gdpS* mutation affects the survival of *S. epidermidis* at low temperature, bacterial cultures in mid-exponential phase were kept at 4℃ for several days, and the optical density at 600 nm was monitored each day after thorough shaking. As illustrated in Fig. 3, compared with the wild-type strain, OD_600_ values of the *gdpS* mutant declined sharply during storage at 4℃, indicating that massive cell death and autolysis occurred in the bacterial cultures. The complementation strain had almost restored survival to the wild-type level at low temperature, while the empty vector control strain displayed a phenotype similar to that of the *gdpS* mutant. These findings suggest that *gdpS* mutation increased the susceptibility of *S. epidermidis* to low temperature.

### Transcriptome changes induced by loss of *gdpS* function

**Fig. 4:**
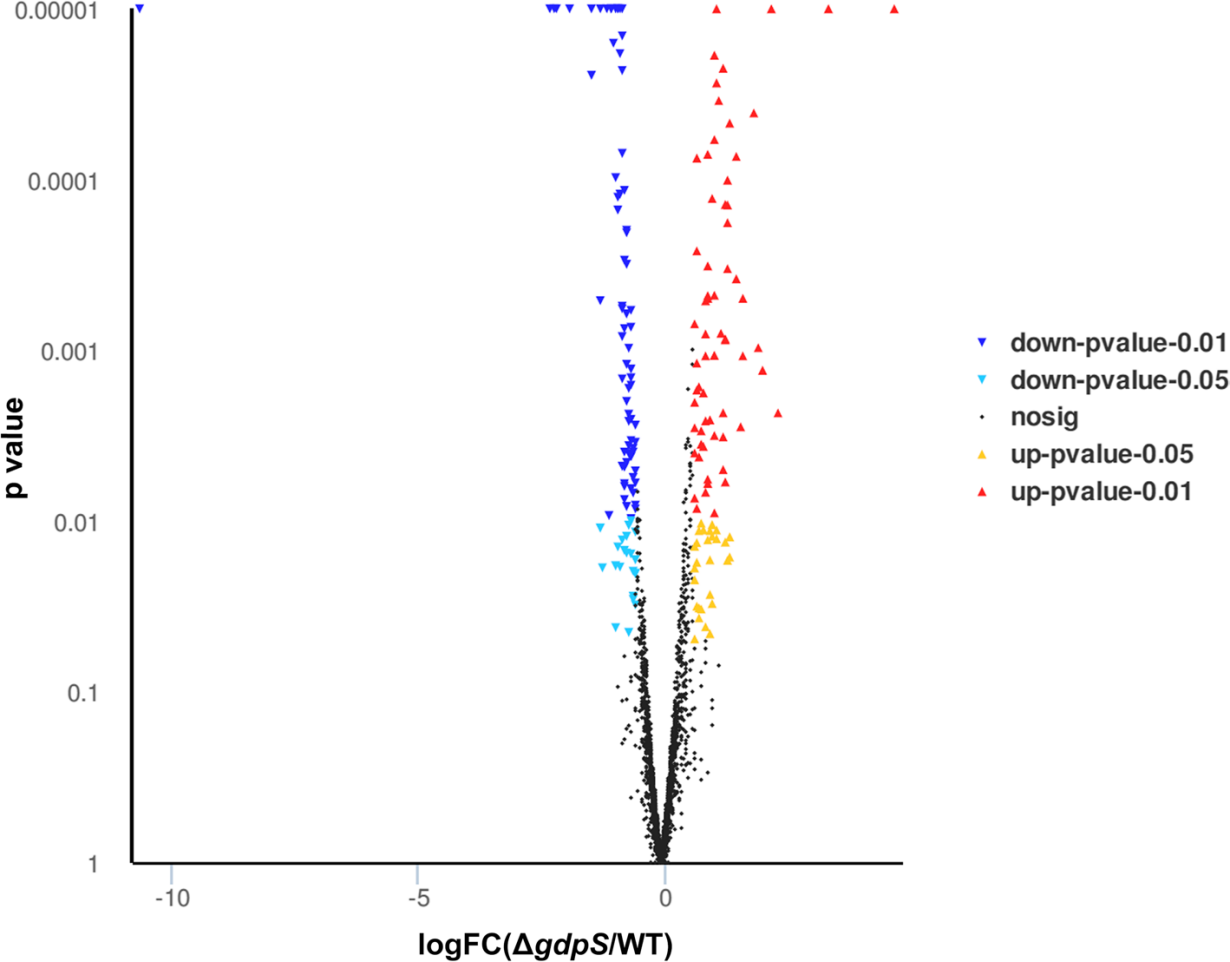
A volcano plot revealing the differences in gene expression between the Δ*gdpS* strain and the wild-type strain. Genes with |log2FC|> = 0.58496 and adjusted *p*-value < 0.05 were considered differentially expressed. Each point represents one gene: dark dots are non-DEGs, red and blue dots are upregulated and downregulated genes, respectively

**Fig. 5:**
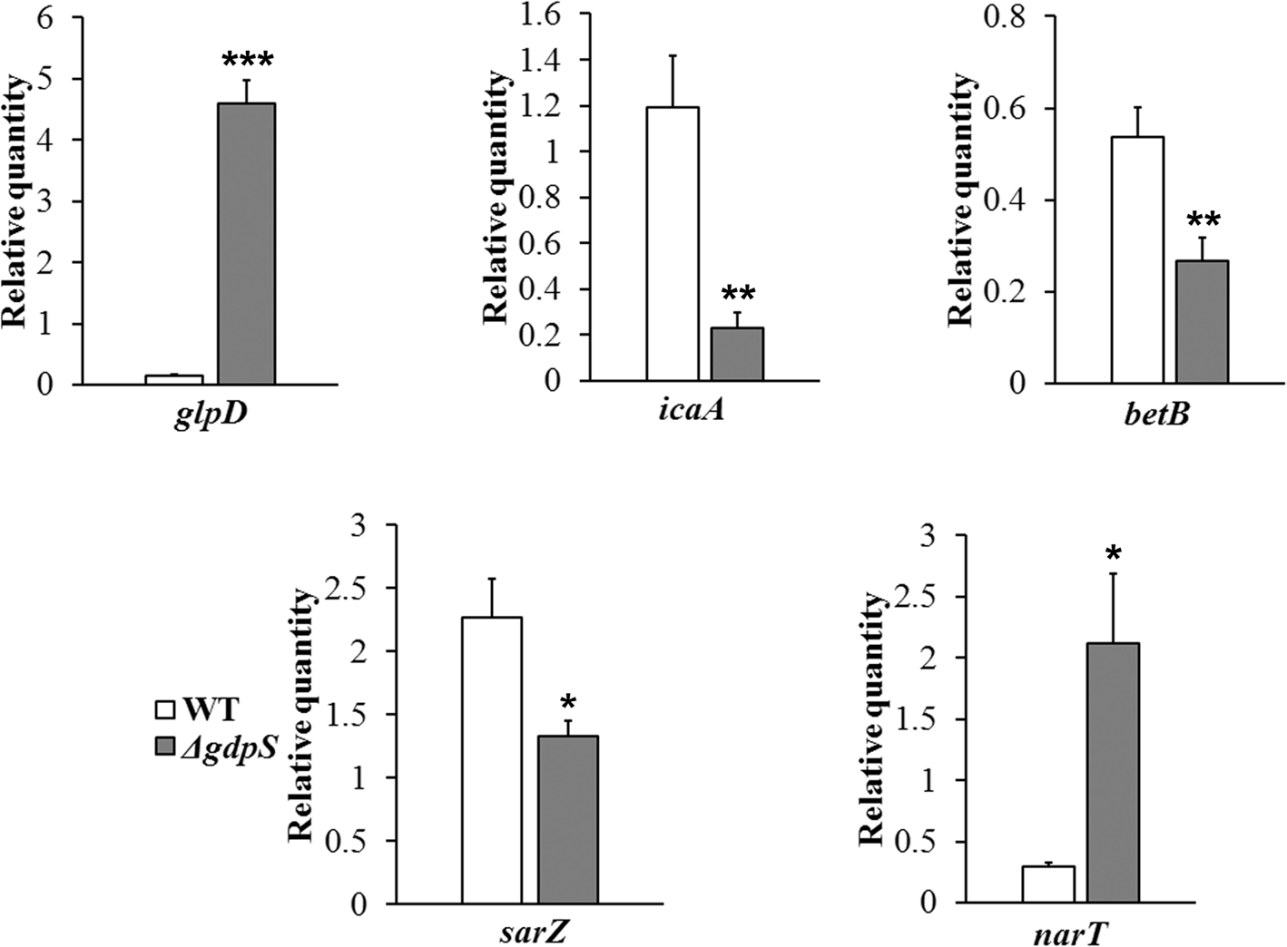
Validation results of RNA-seq profiles by qPCR. Data are means ± SEM of three independent experiments with three replicates. *, *p* < 0.05; **, *p* < 0.01; ***, *p* < 0.001; Δ*gdpS*, vs. wild-type (WT)

To assess the global impact of losing *gdpS* function on *S. epidermidis* physiology, RNA sequencing was performed to compare the gene expression profile between the *gdpS* mutant and the wild-type strain. As a result, a total of 188 DEGs (differentially expressed genes) with fold change >  = 1.5 was found between the *gdpS* mutant and its parent strain. Of these, 92 genes were significantly upregulated, and 96 genes were downregulated in the *gdpS* mutant relative to the wild-type strain (Fig. 4). To evaluate the reliability of the RNA-seq data, five randomly selected DEGs were analyzed by qRT-PCR, using the *gyrB* gene as reference. The expression level of five DEGs obtained by qRT-PCR were consistent with the results from RNA-seq (Fig. 5), indicating that the data generated by RNA-seq could be used to further investigate the expression of specific genes.

**Fig. 6:**
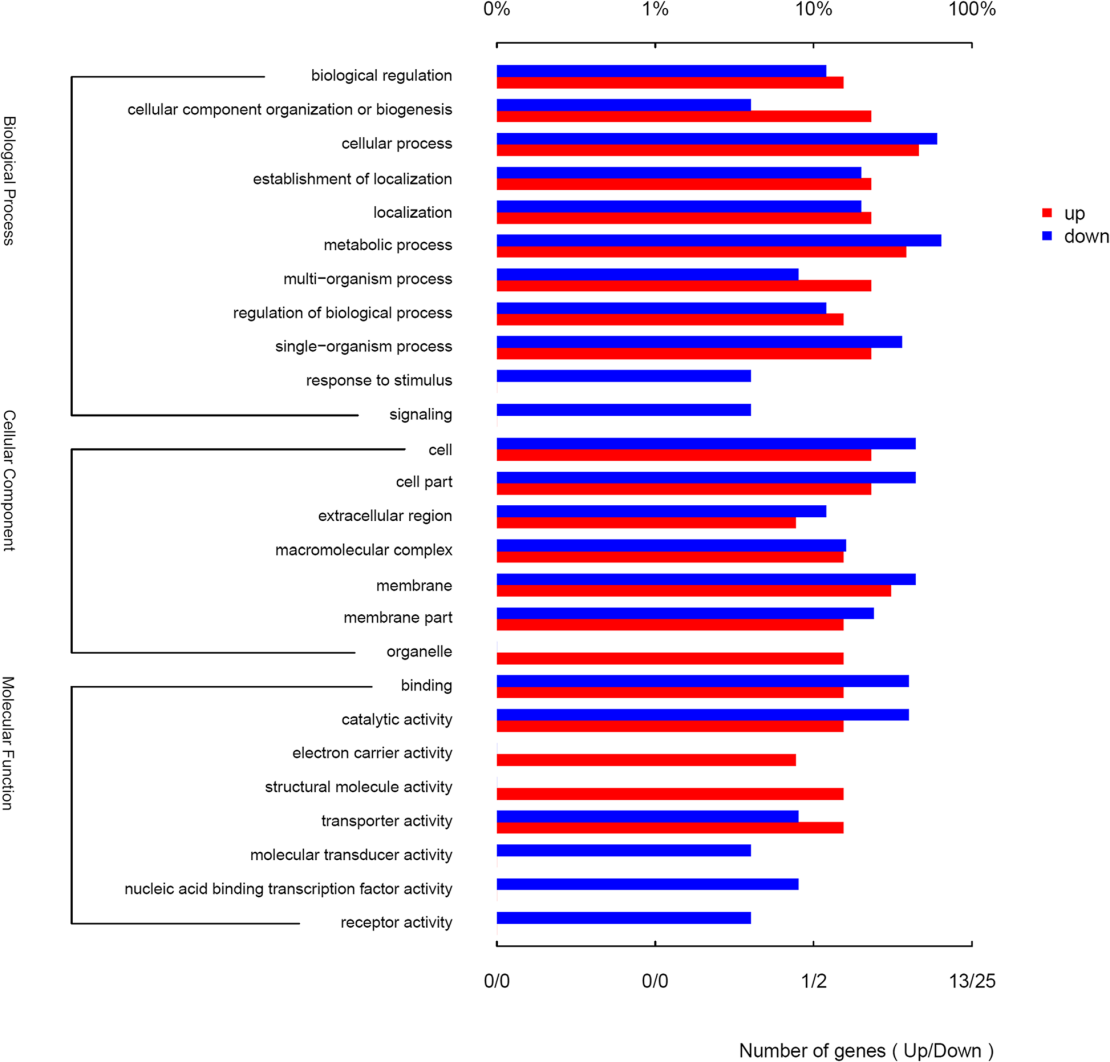
Gene ontology classification of differentially expressed genes (DEGs). The x-axis is the name of category and the y-axis is the number of genes. Red and green denote the upregulated and downregulated genes, respectively

In order to investigate the functions of DEGs, they were assigned by the GO (gene ontology) databases. A total of 38 DEGs were successfully annotated with three main GO terms, of which 32 DEGs were categorized into the ‘biological process’, 24 DEGs into the ‘cellular component’, and 27 DEGs into the ‘molecular function’ (Fig. 6). Among biological processes, the dominant GO terms were ‘cellular process’, ‘metabolic process’ and ‘single-organism process’. For cellular component, ‘cell’, ‘cell part’ and ‘membrane’ were major GO terms. Under the category of molecular function, ‘catalytic activity’ and ‘transporter activity’ were the dominant terms.

### Metabolic pathways are differentially regulated in the *gdpS* mutant strain

The most highly upregulated genes in the *gdpS* mutant are involved in glycerol metabolism, including *glpF, glpK* and *glpD*, with fold changes of 9.9, 4.5 and 25, respectively (Table 1). The *glpF* gene encodes a glycerol facilitator belonging to the aquaporin family of passive transporters. The *glpF* gene usually forms an operon together with the *glpK* gene, which encodes a glycerol kinase phosphorylating glycerol to glycerol-3-phosphate (G3P). The *glpD* gene encodes a membrane-bound, aerobic G3P dehydrogenase. This enzyme is a flavoprotein that catalyzes the oxidation of G3P to dihydroxyacetone-phosphate (DHAP). The oxidation is coupled to the reduction of ubiquinone or menaquinone of the respiratory electron transport chain. DHAP is subsequently catabolized through the Embden-Meyerhof-Parnas (EMP) pathway. In the presence of the above three genes, glycerol can be aerobically utilized by bacteria as a carbon source. The uptake of glycerol into bacterial cells is achieved by facilitated diffusion mediated by GlpF. It is converted by GlpK to G-3-P, which is then dehydrogenated by GlpD to yield DHAP [22–24]. In addition to participating in glycerol metabolism, these genes have also been implicated in formation of persister cell in *Escherichia coli*, *Pseudomonas aeruginosa* and *S. aureus* [25–27]. The persister cells have been defined along with VBNC cells as two dormancy strategies that cope with adverse environments. Previous studies have shown that VBNC cells are not only present with persister cells at much higher numbers in exponential phase but also withstand antibiotic treatment alongside persister cells. Therefore, the two survival modes are viewed as a continuum between actively growing and dead cells, with VBNC cells being in a deeper dormancy depth than persister cells [28, 29]. It is plausible that *gdpS* mutation might cause *S. epidermidis* to enter a VBNC state by increasing expression of genes involved in glycerol metabolism. This warrants further investigation.

**Table 1:** Main genes affected by *gdpS* mutation in *S. epidermidis* (RNA-seq)

locus_tag	Gene symbol	Gene Product	logFC (Δ*gdpS* /WT)	Biological functions/pathways
SERP_RS02180	*gdpS*	GGDEF domain-containing protein	-10.65	
SERP_RS05895		hypothetical protein	1.01	
SERP_RS06915		glutamate ABC transporter permease	1.04	
SERP_RS08330	*ilvC*	ketol-acid reductoisomerase	1.34	Valine, leucine and isoleucine biosynthesis
SERP_RS08335	*leuA*	2-isopropylmalate synthase	1.03	
SERP_RS08340	*leuB*	3-isopropylmalate dehydrogenase	1.1	
SERP_RS09425	*ssaA*	secretory antigen precursor SsaA	-1.02	
SERP_RS09875	*narT*	NarK/NasA family nitrate transporter	1.81	Nitrogen metabolism
SERP_RS09885	*nreB*	sensor histidine kinase	1.28	
SERP_RS09890	*nreA*	nitrate respiration regulation accessory nitrate sensor NreA	1.02	
SERP_RS09895	*narI*	respiratory nitrate reductase subunit gamma	1.24	
SERP_RS09900	*narJ*	respiratory nitrate reductase subunit delta	1.6	
SERP_RS09905	*narH*	respiratory nitrate reductase subunit beta	1.59	
SERP_RS09910	*narG*	respiratory nitrate reductase subunit alpha	1.54	
SERP_RS09915	*cobA*	uroporphyrinogen-III C-methyltransferase	1.15	
SERP_RS09920	*nirD*	nitrite reductase small subunit	1.26	
SERP_RS09925	*nirB*	nitrite reductase large subunit	1.28	
SERP_RS10790	*betB*	betaine aldehyde dehydrogenase	-1	
SERP_RS11285	*icaA*	poly-beta-1,6 N-acetyl-D-glucosamine synthase	-2.33	Pathogenesis
SERP_RS11290	*icaD*	poly-beta-1,6-N-acetyl-D-glucosamine synthesis protein IcaD	-2.24	
SERP_RS11295	*icaB*	poly-beta-1,6-N-acetyl-D-glucosamine N-deacetylase	-2.19	
SERP_RS11300	*icaC*	poly-beta-1,6-N-acetyl-D-glucosamine export protein	-1.93	
SERP_RS11375	*mqo*	malate:quinone oxidoreductase	1.04	
SERP_RS11730	*gehD*	YSIRK domain-containing triacylglycerol lipase GehD	-1.49	
SERP_RS12370	*rlmH*	23S rRNA (pseudouridine(1915)-N(3))-methyltransferase RlmH	-1.32	
SERP_RS01900		hypothetical protein	-1.16	
SERP_RS01910	*fruR2*	DeoR/GlpR family DNA-binding transcription regulator	-1.08	
SERP_RS01960		DM13 domain-containing protein	-1.1	
SERP_RS02070	*adhE*	bifunctional acetaldehyde-CoA/alcohol dehydrogenase	-1.5	
SERP_RS02810	*argH*	argininosuccinate lyase	1.23	Arginine biosynthesis
SERP_RS02815	*argG*	argininosuccinate synthase	1.92	
SERP_RS02920	*oppB*	ABC transporter permease	1.02	ABC transporter
SERP_RS02925	*oppC*	ABC transporter permease	1.2	
SERP_RS02930	*oppD*	ABC transporter ATP-binding protein	1.21	
SERP_RS03730	*psmβ1a*	beta-class phenol-soluble modulin	1.44	Pathogenesis
SERP_RS03740	*psmβ3*	beta-class phenol-soluble modulin	1.3	
SERP_RS03870	*pyrP*	uracil permease	1.23	
SERP_RS03895	*pyrF*	orotidine-5'-phosphate decarboxylase	1.17	Pyrimidine metabolism
SERP_RS03900	*pyrE*	orotate phosphoribosyltransferase	1.22	
SERP_RS04360	*glpF*	glycerol uptake facilitator protein	3.32	Glycerophospholipid metabolism
SERP_RS04365	*glpK*	glycerol kinase	2.16	
SERP_RS04370	*glpD*	aerobic glycerol-3-phosphate dehydrogenase	4.66	
SERP_RS04825	*lysC*	aspartate kinase	1.47	Lysine biosynthesis
SERP_RS04830	*asd*	aspartate-semialdehyde dehydrogenase	1.26	
SERP_RS04835	*dapA*	4-hydroxy-tetrahydrodipicolinate synthase	1.17	
SERP_RS04840	*dapB*	4-hydroxy-tetrahydrodipicolinate reductase	1.07	

In *Borrelia burgdorferi*, the causative agent of Lyme disease, expression of the *glpFKD* operon is essential for fitness of the spirochetes in ticks, which can produce glycerol as a cryoprotective molecule. It has been shown that c-di-GMP can positively regulate the *glpFKD* operon via the c-di-GMP effector PlzA. Moreover, in the absence of c-di-GMP, PlzA also can function as a negative regulator of *glpFKD* expression [30–32]. In the present study, transcriptomic data indicates *gdpS* negatively controls expression of the *glpFKD* gene cluster in *S. epidermidis*. In addition, inactivation of *gdpS* in *S. aureus* results in upregulation of *glpT* gene encoding a G3P transporter, as revealed by microarray data [14]. These findings imply that while *gdpS* involvement in c-di-GMP synthesis in *Staphylococci* is under debate, the regulatory role of the GGDEF-containing gene in glycerol metabolism may be highly conserved.

**Fig. 7:**
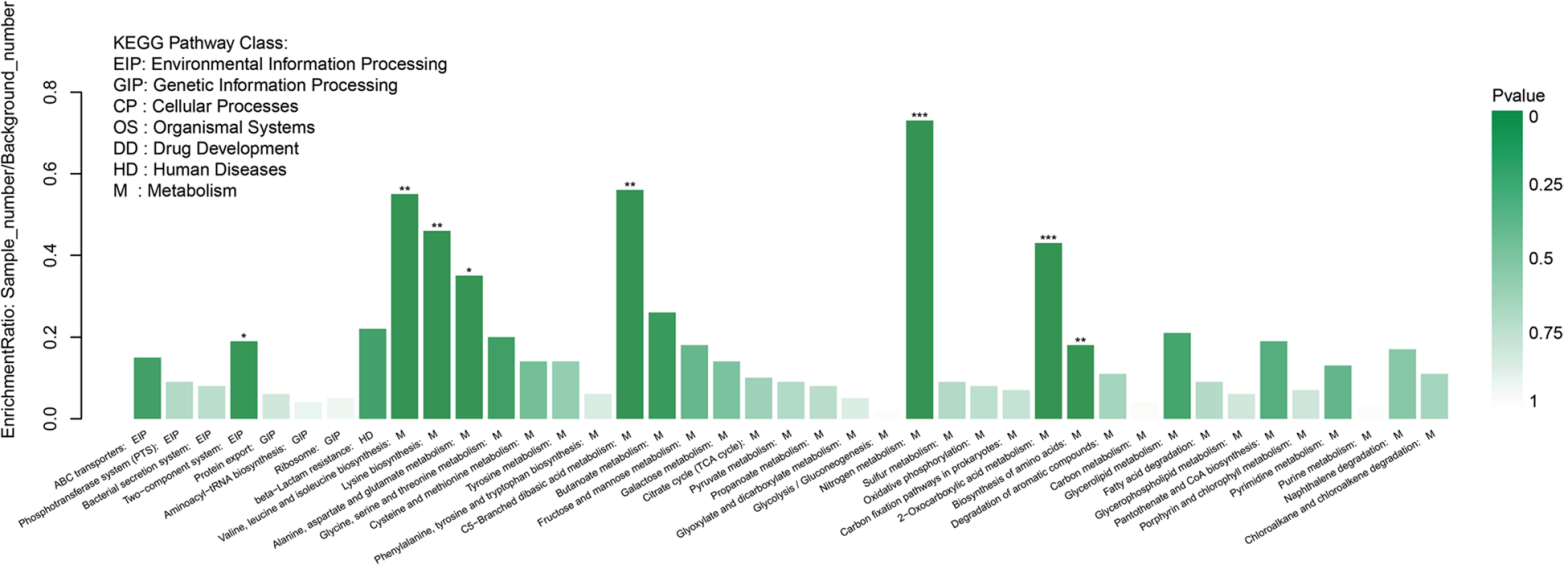
Functional categories and Kyoto Encyclopedia of Genes and Genomes (KEGG) enrichment analysis of DEGs using the Majorbio cloud platform (*p* < 0.01)

The KEGG pathway enrichment analysis revealed that the most affected genes in the *gdpS* mutant are those involved in nitrogen metabolism (Fig. 7, Supplementary File 1), including *narT* gene and *narABC*, *narGHJI* and *nirBD* operons, with almost 2- to threefold increased expression (Table 1). The *narT* gene encodes a transport protein that is required for nitrate uptake and nitrite export [33]. The *narGHJI* operon encodes a membrane-bound respiratory nitrate reductase that is responsible for generation of nitrite [34]. Under anaerobic conditions, nitrite can be further reduced to ammonium by the *nirBD* encoded cytosolic NADH-dependent nitrite reductase [35]. In contrast to nitrate reduction, NirBD-catalyzed nitrite dissimilation is not coupled to the formation of proton motive force and, hence, is not a respiratory pathway. It serves rather to detoxify the nitrite that accumulates in nitrate-consuming cells and as an electron sink to reproduce NAD^+^ [36, 37]. The three-cistron operon *nreABC* has been identified to encode an oxygen sensing two-component system NreB/NreC and a nitrate receptor NreA. When oxygen is depleted, autophosphorylation activity of the cytoplasmic histidine kinase NreB increases in the presence of nitrate and NreA. The response regulator NreC is subsequently phosphorylated by NreB and specifically binds to the promoters of the *narT* gene and of the *narGHJI* and *nirBD* operons to activate transcription [37–39]. Since previous studies have shown that both aerobic and anaerobic G3P dehydrogenase can transfer hydrogens from G3P to nitrate, leading to growth of *E. coli* and *S. aureus* on glycerol as the carbon source and nitrate as the hydrogen acceptor [40, 41]. We speculate the *gdpS* gene may negatively modulate the entire respiratory pathway consisting of glycerol catabolism and nitrate reduction in *S. epidermidis*.

It is noteworthy that the *betB* gene encoding betaine-aldehyde dehydrogenase was downregulated approximately twofold in the *gdpS* mutant. Under the catalysis of this gene product, betaine-aldehyde can be oxidized to glycine betaine (GB). GB has been widely studied as an excellent osmoprotectant in both the prokaryotic and eukaryotic world [42]. Recently, GB has also been reported to confer tolerance against low temperature in bacteria and plants by preventing cold-induced aggregation of cellular proteins and maintaining an optimum membrane fluidity [43–45]. In the present study, loss of *gdpS* function resulted in increased susceptibility of *S. epidermidis* to low temperature, which led us to speculate that *gdpS* may promote tolerance of *S. epidermidis* to low temperature through regulating the expression of *betB* gene responsible for glycine betaine biosynthesis.

### Genes involved in pathogenesis are altered in the *gdpS* mutant strain

**Fig. 8:**
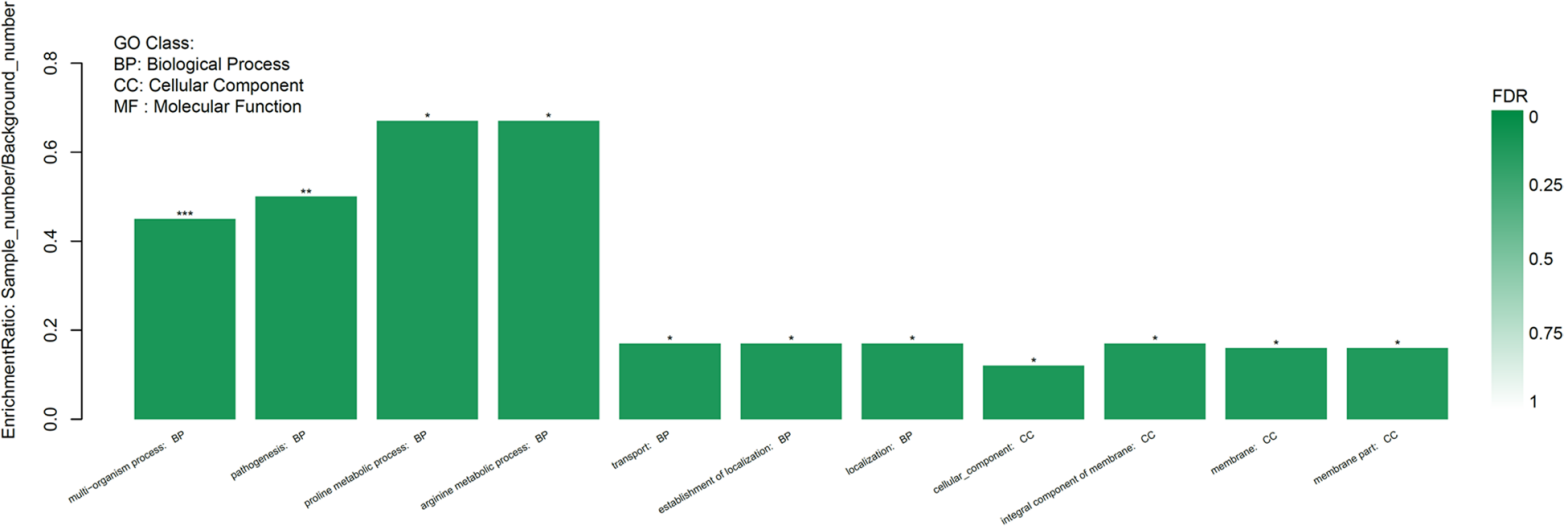
Functional categories and gene Ontology (GO) enrichment analysis of DEGs using the Majorbio cloud platform. Seventy-three terms were identified; the first 5 enrichment terms of upregulated and downregulated genes are shown based on the *P* values from low to high, respectively; *p* < 0.05

Apart from multi-organism process, pathogenesis is the significantly enriched GO term (Fig. 8, Supplementary File 2). Under this category, the *ica* operon involved in PIA biosynthesis and the *psmβ* operon encoding phenol-soluble modulins (PSMs) are included. It has been demonstrated by Holland et.al and our group that the *gdpS* gene promotes biofilm formation in an *ica*-dependent manner [15]. Transcriptional profiling analysis in this study discloses the *ica* operon as the most remarkably downregulated genes in the *gdpS* mutant. Also the beta subclass of PSMs has also been proposed to promote *S. epidermidis* biofilm mature and dissemination by mediating biofilm cluster detachment through its surfactant-like property [46]. In the present study, transcript levels of the three genes, *psmβ1a*, *psmβ1b* and *psmβ3*, which constitute part of the four-gene *psmβ* operon, increased approximately two-fold in the *gdpS* mutant. Therefore, the transcriptomic data imply that *gdpS* might enhance biofilm formation in *S. epidermidis* not only by elevating expression of *ica* operon but also repressing transcription of *psmβ* operon.

## Conclusion

This study explored more physiological roles of *gdpS* gene apart from enhancing biofilm formation in *S. epidermidis*. These include that it may participate in formation of presumed VBNC state under induction of cryopreservation and confer *S. epidermidis* tolerance against low temperature. Transcriptome experiments displayed that this may be attributed to its influence on expression of genes related to energy metabolism and synthesis of osmoprotectant.

## Materials and methods

### Bacterial strains and growth conditions

The *gdpS* deletion mutant strain (Δ*gdpS*) of *S. epidermidis* 35984 M (WT) and its complementation strain C-*gdpS*, vector control strain C-pCN (Δ*gdpS* complemented with the empty vector pCNcat) were constructed in our laboratory previously [16]. All the strains were maintained as glycerol (40%, v/v) stocks, which were prepared after growth to mid-exponential phase (approximately 4 h), and were frozen at − 80 °C. For liquid cultivation, overnight cultures of *S. epidermidis* from each single colony were inoculated respectively into TSB medium (BD Difco) at a dilution of 1:100 with a ratio of flask volume to medium volume of 5:1. The subcultures were grown to mid-exponential phase (approximately 4 h) under 200 rpm agitation at 37 °C. When necessary, chloramphenicol at a final concentration of 10 μg/mL was added. For solid cultivation, an aliquot of 5 μL of glycerol stocks or serially diluted cultures was spotted onto the TSB agar plate or Columbia blood agar plate and incubated at 37 °C for about 24 h. The bacterial colonies on the agar plates were photographed.

### Susceptibility to low temperature

To detect the susceptibility of the *gdpS* mutant strain to low temperature, overnight cultures of *S. epidermidis* strains were diluted into fresh TSB medium and grown to logarithmic phase (4 h, OD_600_ = 2) at 37 °C. Bacterial cultures were kept at 4 °C for four days and the turbidity was measured at 600 nm each day.

### RNA extraction

For transcriptome sequencing, all the *S. epidermidis* strains were grown to mid-exponential phase in 6 mL TSB medium under the conditions described above. Prior to RNA isolation, two volumes of RNAprotect Bacteria Reagent (Qiagen, cat#76,506) were added to one volume of bacterial culture to provide immediate stabilization of RNA. Thereafter, bacterial cells were pelleted by centrifugation at 5000 g for 10 min and vortexed with 0.9 mL Buffer RLT plus 1 mL Ziconia-silica beads (0.1 mm diameter). The bacterial sediments were then mechanically disrupted on a Mini-Beadbeater (Biospec) at maximum speed four times for 40 s with intermittent cooling on ice. The subsequent RNA extraction using RNeasey Mini kit (Qiagen, cat#74,104) were performed according to the manufacturer’s instruction. To eliminate any genomic DNA contamination, DNase digestion in solution and on-column DNase digestion were both carried out using RNase-Free DNase Set (Qiagen, cat#79,254) as recommended by the manufacturer during RNA extraction. The total RNA was eventually eluted from the column with 60 μL of RNase-free water.

### Transcriptome sequencing

For genome-wide RNA sequencing, total RNA of each sample was subsequently submitted to Majorbio Co., Ltd. (Majorbio, Shanghai, China). The quality of the total RNA was assessed using a 2100 Bioanalyzer (Agilent, USA) and its amount was quantified using an ND-2000 instrument (NanoDrop Technologies, USA). Afterward, ribosomal RNA was removed from the total RNA using the Ribo-Zero magnetic kit (Epicentre, USA) and the yielded mRNA was chemically fragmented to approximately 200-nt-long oligonucleotides using fragmentation buffer. The cDNA libraries were then generated from enriched mRNA samples using the Illumina TruSeq RNA sample prep kit as follows. Synthesis of double-stranded cDNA was performed using the SuperScript double-stranded cDNA synthesis kit (Invitrogen, USA) with random hexamer primers (Illumina, USA). Then, the synthesized cDNA was subjected to end repair, A-base addition and adapter ligation according to Illumina’s library construction protocol. The libraries were size-selected for cDNA target fragments of 200 bp on 2% low-range ultra-agarose followed by PCR amplification using Phusion DNA polymerase (NEB, USA) for 15 PCR cycles. After quantification by TBS-380, the paired-end RNA-seq library was sequenced with the Illumina HiSeq4000 platform (2 × 150-bp read length).

### Bioinformatics analysis

The trimming and quality control of raw end-paired reads were performed using SeqPrep (https://github.com/jstjohn/SeqPrep) and Sickle (https://github.com/najoshi/sickle) with default parameters. Clean data from each sample were then aligned to the genome of *S.epidermidis* strain ATCC35984 (NCBI Reference Sequence: NC_002976.3) using Bowtie2 (http://www.bowtie-bio.sourceforge.net/bowtie2/manual). To estimate the expression level of each gene, FPKM (fragments per kilobase of transcript per million fragments) was calculated using RSME (http://deweylab.biostat.wisc.edu/rsem/). The differentially expressed genes (DEGs) were analyzed with EdgeR (http://www.bioconductor.org/packages/2.12/bioc/html/edgeR.html) by comparing the transcript abundance between the *gdpS* mutant and its parent strain. The DEGs were selected using the following filter criteria: an adjusted *p* value < 0.05 and fold change >  = 1.5 (|log2FC|> = 0.58496). The GO and KEGG pathway enrichment analysis for the DGEs were conducted by Goatools (https://github.com/tanghaibao/Goatools) and KOBAS (http://kobas.cbi.pku.edu.cn/home.do) [47, 48].

### Experimental validation of RNA-seq profiles by qPCR

To confirm the reliability of the transcriptome data, five DEGs namely *icaA*, *glpA*, *sarZ*, *saeS* and *narT* were chosen for qRT-PCR validation. The cDNA was synthesized from the same RNA samples used for RNA-seq by using the GoScript™ Reverse Transcription Mix, Random Primers (Promega) according to the manufacturer’s instructions. The qPCR reactions were performed using the FastStart Essential DNA Green Master (Roche, USA) on a LightCycler® 96 instrument (Roche, USA). The PCR amplification program was set as follows: initial denaturation at 95 °C for 10 min, followed by 40 cycles of 95 °C for 15 s, 60 °C for 15 s. Melting curve analysis was monitored at the end of the PCR amplification by first heating to 95 °C for 10 s, cooling to 65 °C for 60 s, and then melting with continuous acquisition (5 readings/°C) of fluorescence signal until 97 °C. Each reaction was performed in triplicate, and the *gyrB* gene was employed as internal control for normalization in the assay. The relative expression levels of target genes were calculated using the 2^−△△Ct^ method and compared with the results of RNA-seq analysis. All primer pairs for five selected genes and the *gyrB* gene were listed in Supplementary file 3.

## Statistical analysis

All experiments were performed in triplicate or separately reproduced three times. Student’s t test on the VassarStats Web site was used to compare data between two groups. A *p* value of < 0.05 indicated that there were significant differences between groups.

## Supplementary Information

**Additional file 1: Table S1.** List of KEGG pathways that were enriched with differentially expressed genes (DEG) detected between the Δ*gdpS* strain and the wild-type strain.

**Additional file 2: Table S2.** List of Gene Ontology (GO) terms that were enriched with differentially expressed genes (DEG) detected between the Δ*gdpS* strain and the wild-type strain.

**Additional file 3: Table S3.** Primers used in this study.

## Data Availability

The raw data for the transcriptome sequencing of *S. epidermidis* 35984 M and the *S. epidermidis* Δ*gdpS* mutant have been deposited in the NCBI Sequence Read Archive (SRA) database under BioProject accession number PRJNA865168.
