# Supplementary material for: Mutation of gdpS gene induces a viable but non-culturable state in Staphylococcus epidermidis and changes in the global transcriptional profile
Source: BMC Microbiol. 2022 Dec 1;22:288. doi: 10.1186/s12866-022-02708-6 (PMC9714401; doi:10.1186/s12866-022-02708-6)
Supplement: Supplementary file 3 — Additional file 3: Table S3. Primers used in this study. [file 12866_2022_2708_MOESM3_ESM.docx]

Table S3 Primers used in this study

| Primer name | Sequence | Product size |
| --- | --- | --- |
| *gyrB*-RF | CACCGTGAAGACCGCCAGATAC | 99 |
| *gyrB*-RR | AGATGGGACGCCCTGCTGTC |  |
| *glpD*-RF | GGTGGTGACGTTGGCGGAAG | 100 |
| *glpD*-RR | AGCGACGTGCCACATCTTCATC |  |
| *sarZ*-RF | TCTTGGATTCTGGCACGCTG | 90 |
| *sarZ*-RR | TCCGTTCATCTTGTTCTTCACGAG |  |
| *sarX*-RF | GTTGACGTTAAACGATCTTGCTGT | 102 |
| *sarX*-RR | AACTCTCCTGTAGCCAGTTTTAGA |  |
| *narT*-RF | CCGGGTGATTGAGCTTGACC | 84 |
| *narT*-RR | TGTAGGAGCGAAATGGGTGT |  |
| *icaA*-RF | ACGAACCACGTGCTCTATGC | 82 |
| *icaA*-RR | CCTTGAGCCCATCGAACCCT |  |
| *betB-RF* | CGGTGTGCTGTTGAGATAACTGGTC | 150 |
| *betB-RR* | AGTGTGCTCTGCTGGTTCAAGAATC |  |
